# Supplementary material for: Patterns of Intron Gain and Loss in Fungi
Source: PLoS Biol. 2004 Nov 30;2(12):e422. doi: 10.1371/journal.pbio.0020422 (PMC532390; doi:10.1371/journal.pbio.0020422)
Supplement: Table S1 — Also available at http://genes.mit.edu/NielsenEtAl/. (4.3 MB ZIP). [file pbio.0020422.st001.zip › NielsenEtAl/html/1427.html]

AN1892.1.NCU05623.1.MG04228.1.FG06415.1


```
 CLUSTAL W (1.82) Multiple Sequence Alignments - Introns Inserted


Sequence 1: NCU05623.1	155 aa
Sequence 2: FG06415.1	152 aa
Sequence 3: MG04228.1	198 aa
Sequence 4: AN1892.1	153 aa
Alignment Length: 199 aa
Number Identitical Residues: 115 aa
Alignment Score (without introns) 5095


MG04228.1 	MDHTRDPCPWVILNDFGGAFCMG0AIGGTIWHGVKGFRNSPYGERRIGALTAIKMRAPVL
NCU05623.1	MDHTRDPCPWVILNDFGGAFAMG0AIGGTIWHGIKGFRNSPYGERRIGAITAIKMRAPAL
FG06415.1 	MDHGRDPCPYVILNDFGGAFCMG0AIGGTIWHGIKGFRNSPYGERRIGAITAIKMRAPVL
AN1892.1  	MDHSRDPCPWVALSDFGGAFCMG0AIGGAVWHGVKGFRNSPYGERRIGAITAIKARAPVL
          	*** *****:* *.******.** ****::***:***************:**** ***.*

MG04228.1 	GGNFG~VWGGLFSTFDCAVKGIR-KKEDPYNAI~IAGFFTGGSLAIRGGYKAARNNAIGC
NCU05623.1	GGNFG1VWGGLFSTFDCAIKGLRNHKEDPWNSI~LAGFFTGGALAVRGGYKAARNGAIGC
FG06415.1 	GGNFG~VWGGLFSTFDCAVKGVR-QKEDPYNAI~IAGFFTGGSLAIRGGYKAARNGAIGC
AN1892.1  	GGNFG~VWGGLFSTFDCAVKGIR-KKEDPYNAI1IAGFFTGGSLAIRGGYKAARNGAIMC
          	***** ************:**:* :****:*:* :*******:**:*********.** *

MG04228.1 	AILLGVIEGVGIGFQKMMAGSTKLE0KPSRSRHKSPPESSTTIQQTNDRRSPLYSPFQAT
NCU05623.1	AVLLAVIEGVGIGFQKMLAGATKLE0APA-----PPPSNEKVLA----------------
FG06415.1 	AVLLAVIEGVGIGFSKMLAGSTKLE~APQ-----PPP-QEATL-----------------
AN1892.1  	AVFLAVIEGVGIGFQRMMADQTKLE0LPP-----APPSDKAVA-----------------
          	*::*.*********.:*:*. ****  *      .**... .                  

MG04228.1 	TSAAAVVYFLGHLWMVGVVSSVL
NCU05623.1	-----------------------
FG06415.1 	-----------------------
AN1892.1  	-----------------------
          	
```
